# Pediatric COVID-19 and Pericarditis Presenting With Acute Pericardial Tamponade

**DOI:** 10.1177/2150135120949455

**Published:** 2020-09-10

**Authors:** Tia T. Raymond, Ashima Das, Shai Manzuri, Stuart Ehrett, Kristine Guleserian, Javier Brenes

**Affiliations:** Division of Cardiac Critical Care, Department of Pediatrics, Medical City Children’s Hospital, Dallas, TX, USA; Division of Pediatric Infectious Disease, Department of Pediatrics, Medical City Children’s Hospital, Dallas, TX, USA; Department of Congenital Heart Surgery, Medical City Children’s Hospital, Dallas, TX, USA

## Abstract

We describe a seven-year-old female with acute pericarditis presenting with pericardial tamponade, who screened positive for coronavirus disease 2019 (COVID-19 [SARS-CoV-2]) in the setting of cough, chest pain, and orthopnea. She required emergent pericardiocentesis. Due to continued chest pain and orthopnea, rising inflammatory markers, and worsening pericardial inflammation, she underwent surgical pericardial decortication and pericardiectomy. Her symptoms and pericardial effusion resolved, and she was discharged to home 3 days later on ibuprofen and colchicine with instruction to quarantine at home for 14 days from the date of her positive testing for COVID-19.

## Introduction

Coronavirus disease 2019 (COVID-19) is a global pandemic with more than 22 million cases worldwide and over 780,000 deaths.^1^ Coronavirus disease 2019 is caused by severe acute respiratory syndrome coronavirus-2 (SARS-CoV-2), resulting in significant morbidity and mortality. Data from China suggest that pediatric COVID-19 cases might be less severe than adults and that children might experience different symptoms than do adults^2,3^; however, disease characteristics among pediatric patients in the United States are continually being described in the literature. Similarly, there is a report of a pediatric patient diagnosed and treated for classic Kawasaki disease in the setting of confirmed COVID-19 infection,^4^ and more recently health officials in the United Kingdom and the United States are warning that COVID-19 could be causing a new and rare immune-mediated inflammatory syndrome termed multisystem inflammatory syndrome in children (MIS-C).^5^

## Case Description

**Figure 1.**
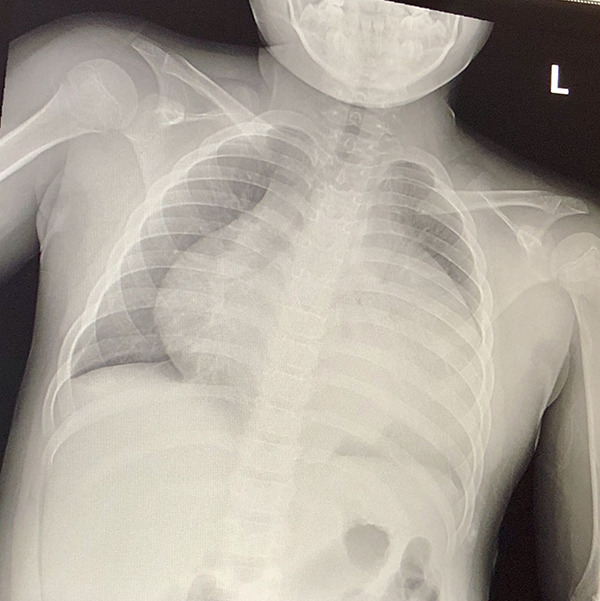
Chest X-ray (CXR) with enlarged cardiac silhouette with bilateral small pleural effusions.

**Figure 2.**
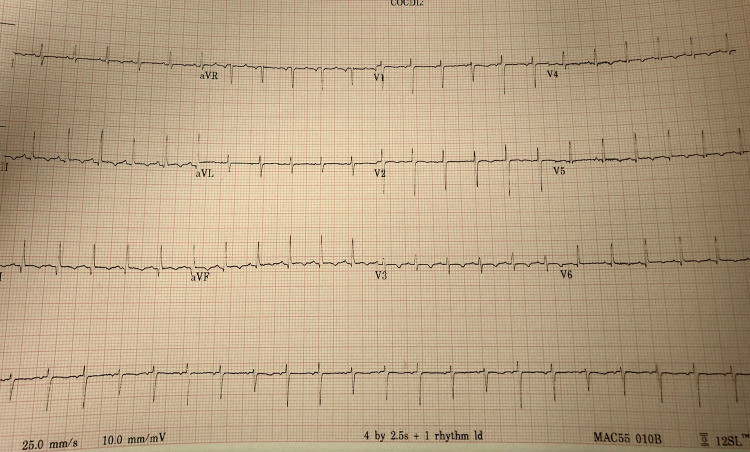
Electrocardiogram (EKG) with sinus tachycardia, T-wave inversion in inferior and lateral leads, and low voltage QRS with electrical alternans.

The patient is a previously healthy seven-year-old female who presented to the emergency room with a 3-day history of cough, chest pain, and orthopnea. She had no history of fever, travel, or exposure to contacts with COVID-19. Vital signs were within normal limits except for tachycardia of 145 beats/min. Examination showed a nontoxic appearing female. A chest X-ray showed an enlarged cardiac silhouette with bilateral small pleural effusions (Figure 1). An electrocardiogram showed sinus tachycardia, T-wave inversion in inferior and lateral leads, and low voltage QRS with electrical alternans (Figure 2). Laboratory evaluation included leukocytosis (17.6 K/µL), thrombocytosis (653 K/mm^3^), microcytic anemia (Hgb 10.7 g/dL, mean corpuscular volume (MCV) 74.0 fL), troponin I 0.01 ng/mL, erythrocyte sedimentation rate (ESR) 43, C reactive protein (CRP) 5.11 mg/dL, B-type natriuretic peptide (BNP) 93 pg/mL, ferritin 134 ng/mL, and negative rapid influenza A/B antigen, *Streptococcus pyogenes* antigen (oropharynx), respiratory virus multiplex polymerase chain reaction (PCR), and a pending nasopharyngeal SARS-CoV-2 PCR. An echocardiogram showed a large circumferential pericardial effusion with right atrial and right ventricular wall collapse suggestive of tamponade physiology, qualitatively normal left ventricular systolic function, and normal coronaries (Supplementary file 1).

**Figure 3.**
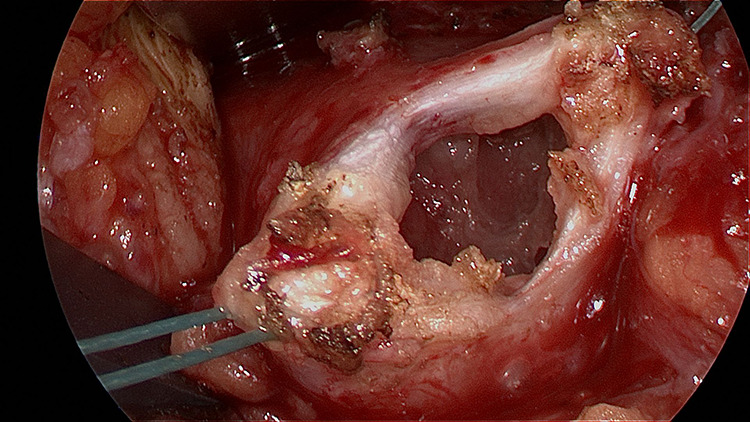
Tissue specimen of the thickened pericardium with significant fibrinous adhesions to the myocardium.

She was emergently transferred to the pediatric cardiac intensive care unit (CICU) where she underwent emergent intubation and pericardiocentesis with pericardial drain placement performed under echocardiographic guidance. Given concerns for possible COVID-19, all procedures were done in full personal protective equipment. Serosanguinous fluid (690 mL) was removed until resolution of the pericardial effusion by echocardiography. The fluid was consistent with a transudate and revealed an elevated protein (6.6 g/dL), red blood cells (137,367/mm^3^), and no evidence of hematolymphoid malignancy with 76.8% neutrophils. Subsequent SARS-CoV-2 PCR (DiaSorin) on admission was negative, and she was initiated on ibuprofen and colchicine without antibiotics given a likely nonbacterial etiology. Workup for pericarditis included negative antinuclear antibodies (ANA) titer, tuberculosis (TB) spot test, PCR for Epstein-Barr virus (EBV), cytomegalovirus (CMV), human herpes virus (HHV) 6, adenovirus, enterovirus, mycoplasma, parvovirus, and HIV. She developed low grade fever (38.1 °C) on admission and had worsening fever (39.6 °C) on hospital day 3 that never returned, for which a repeat SARS-CoV-2 PCR was sent (DiaSorin) which was negative. Given continued pericardial drainage, continued chest pain and orthopnea, rising inflammatory markers (CRP 9.6 mg/dL, ESR 88, BNP 118 pg/mL), evidence of loculated pericardial effusion, inflow variation, and thickened echogenic areas within the pericardium, we proceeded with surgical pericardial exploration with decortication, pericardial biopsy, and pericardiectomy on hospital day 7. Given our CICU policy on routine COVID-19 testing within 48 hours of an aerosolizing procedure, we repeated a nasopharyngeal SARS-CoV-2 PCR (DiaSorin) test which returned positive for a single primer (ORF1ab). Intraoperatively, the pericardium was markedly thickened with fibrovascular adhesions to the myocardium (Figure 3) and tissue samples noted a subacute histologic appearance of a neutrophil-rich fibrinous pericarditis undergoing organization by granulation tissue and fibrosis with reactive mesothelial and stromal changes. Universal PCR testing of the pericardial fluid and pericardial rind for bacteria, fungi, and mycobacteria was negative. The pericardial fluid was negative for SARS-CoV-2 PCR (not approved test), and attempts to send pericardial tissue to the Centers for Disease Control and Prevention (CDC) or Dallas County Health Department for further SARS-CoV-2 testing were denied.

Following the surgical pericardiectomy, she had immediate improvement in her chest pain and orthopnea with resolution of the pericardial effusion and continued good ventricular function and normal coronaries. Her chest drains were removed on postoperative day 2, and she had no reaccumulation of pericardial fluid. A fourth SARS-CoV-2 PCR (DiaSorin) was resent on her surgical day and was again positive for a single primer (ORF1ab), but was negative via a different SARS-CoV-2 PCR platform (Luminex) as requested by infectious disease consultants. Given significant improvement following the surgical pericardiectomy, she was not treated with intravenous immunoglobin (IVIG) for the positive SARS-CoV-2 tests. She was discharged to home on postoperative day 3 on ibuprofen with plans to follow-up with pediatric cardiology for repeat echocardiographic evaluation in two weeks.

## Discussion

Coronavirus disease 2019 has mostly been diagnosed using nasal or pharyngeal swabs or blood specimens that were positive for 2019-nCoV nucleic acid using real-time, reverse transcriptase-polymerase chain reaction assays (RT-PCR). In our patient, the first and second SARS-CoV-2 tests were sent on hospital days 1 and 3, while the patient was awake, via nasopharyngeal swab. It is plausible that an adequate sample was not obtained, given the patient was fully awake and the test requires the nasopharyngeal swab to be inserted deep into the posterior nasopharynx which can be difficult to obtain. The third and fourth SARS-CoV-2 tests, which were positive, were drawn while the patient was sedated and intubated, thus perhaps a more adequate sample from the nasopharynx was obtained. It is also possible that the nasopharyngeal swabs were negative because it was late in the disease process and viral shedding may have been lower in the nasopharynx in comparison to the lower respiratory tract. Additionally, an endotracheal SARS-CoV-2 test may have been helpful, but the third and fourth positive nasopharyngeal swabs did not come back until after she was extubated. Our hospital has two SARS-CoV-2 test platforms because one holds a larger number of samples than the other, allowing tests to be batched. Both platforms have reported 100% sensitivity and specificity. Our patient does meet the CDC case definition of MIS-C in children: <21 years presenting with fever, laboratory evidence of inflammation, and evidence of clinically severe illness requiring hospitalization, with multisystem (>2) organ involvement (cardiac, renal, respiratory, hematologic, gastrointestinal, dermatologic, or neurological); no alternative plausible diagnoses; positive for current or recent SARS-CoV-2 infection by RT-PCR, serology, or antigen test; or exposure to a suspected or confirmed COVID-19 case within the four weeks prior to the onset of symptoms. Finally, an immunoglobulin G (IgG) and IgM antibody test for SARS-CoV-2 could have been helpful in diagnosing remote versus recent infection, but this test was not available in our hospital at the time. Unfortunately, the patient did not show up for follow-up as indicated two weeks after discharge.

The significance of the positive COVID-19 tests remains entirely unclear, however, it is the only pathogen that was identified with an extensive laboratory workup for the etiology of the pericarditis. It is difficult to ascertain whether this was a postinflammatory COVID-19 presentation (ie, MIS-C) versus whether it was an active COVID-19 infection causing pericardial inflammation. As we continue to see the spread of COVID-19 and increase in cases worldwide, the clinical criteria for testing of COVID-19 in pediatrics and the clinical spectrum of disease presentation are yet to be defined.

## Conclusion

The spectrum of clinical presentation patterns of pediatric COVID-19 infections in children continues to be reported, and this case report may serve as a useful reference to other clinicians caring for pediatric patients affected by COVID-19. Clinicians should be aware of the possibility of COVID-19 infection presenting as a potential association with pericarditis and pericardial effusions resulting in pericardial tamponade, with or without myocardial involvement, as well as the newly emerging MIS-C.
